# Spatial mapping and prediction of *Plasmodium falciparum* infection risk among school-aged children in Côte d’Ivoire

**DOI:** 10.1186/s13071-016-1775-z

**Published:** 2016-09-07

**Authors:** Clarisse A. Houngbedji, Frédérique Chammartin, Richard B. Yapi, Eveline Hürlimann, Prisca B. N’Dri, Kigbafori D. Silué, Gotianwa Soro, Benjamin G. Koudou, Serge-Brice Assi, Eliézer K. N’Goran, Agathe Fantodji, Jürg Utzinger, Penelope Vounatsou, Giovanna Raso

**Affiliations:** 1 Unité de Formation et de Recherche Sciences de la Nature, Université Nangui Abrogoua, 02 BP 801, Abidjan 02, Côte d’Ivoire; 2 Département Environnement et Santé, Centre Suisse de Recherches Scientifiques en Côte d’Ivoire, 01 BP 1303, Abidjan 01, Côte d’Ivoire; 3 Department of Epidemiology and Public Health, Swiss Tropical and Public Health Institute, P.O. Box, CH-4002 Basel, Switzerland; 4 University of Basel, P.O. Box, CH-4003 Basel, Switzerland; 5 Unité de Formation et de Recherche Biosciences, Université Félix Houphouët-Boigny, 22 BP 522, Abidjan 22, Côte d’Ivoire; 6 Programme National de Santé Scolaire et Universitaire, 01 BP 1725, Abidjan 01, Côte d’Ivoire; 7 Vector Group, Liverpool School of Tropical Medicine, Liverpool, L3 5QA UK; 8 Institut Pierre Richet de Bouaké, Institut National de Santé Publique, BP 1500, Bouaké, Côte d’Ivoire; 9 Programme National de Lutte contre le Paludisme, Ministère de la Santé et de la Lutte contre le SIDA, BP V 4, Abidjan, Côte d’Ivoire

**Keywords:** Bayesian modelling, Côte d’Ivoire, Malaria, *Plasmodium falciparum*, School-aged children

## Abstract

**Background:**

In Côte d’Ivoire, malaria remains a major public health issue, and thus a priority to be tackled. The aim of this study was to identify spatially explicit indicators of *Plasmodium falciparum* infection among school-aged children and to undertake a model-based spatial prediction of *P. falciparum* infection risk using environmental predictors.

**Methods:**

A cross-sectional survey was conducted, including parasitological examinations and interviews with more than 5,000 children from 93 schools across Côte d’Ivoire. A finger-prick blood sample was obtained from each child to determine *Plasmodium* species-specific infection and parasitaemia using Giemsa-stained thick and thin blood films. Household socioeconomic status was assessed through asset ownership and household characteristics. Children were interviewed for preventive measures against malaria. Environmental data were gathered from satellite images and digitized maps. A Bayesian geostatistical stochastic search variable selection procedure was employed to identify factors related to *P. falciparum* infection risk. Bayesian geostatistical logistic regression models were used to map the spatial distribution of *P. falciparum* infection and to predict the infection prevalence at non-sampled locations via Bayesian kriging.

**Results:**

Complete data sets were available from 5,322 children aged 5–16 years across Côte d’Ivoire. *P. falciparum* was the predominant species (94.5 %). The Bayesian geostatistical variable selection procedure identified land cover and socioeconomic status as important predictors for infection risk with *P. falciparum*. Model-based prediction identified high *P. falciparum* infection risk in the north, central-east, south-east, west and south-west of Côte d’Ivoire. Low-risk areas were found in the south-eastern area close to Abidjan and the south-central and west-central part of the country.

**Conclusions:**

The *P. falciparum* infection risk and related uncertainty estimates for school-aged children in Côte d’Ivoire represent the most up-to-date malaria risk maps. These tools can be used for spatial targeting of malaria control interventions.

**Electronic supplementary material:**

The online version of this article (doi:10.1186/s13071-016-1775-z) contains supplementary material, which is available to authorized users.

## Background

Malaria is a vector-borne disease that is widespread in sub-Saharan Africa. In 2015, an estimated 188 million malaria cases and 395,000 deaths occurred in Africa [1]. According to the Global Burden of Disease study, malaria is responsible for 31.7 million years lived with disability (YLDs) [2]. Malaria also drains the social and economic development of affected countries [3–5]. Over the past 15 years, malaria control interventions have averted 663 million clinical cases across Africa [6].

For the implementation of best-practice control strategies and intervention planning, in-depth knowledge of spatial characteristics and factors that influence malaria is needed. Predictive risk mapping has proven to be an important tool for malaria control [7, 8]. In particular, the use of remote sensing technologies, coupled with geographic information system (GIS) allows to link high-resolution environmental data to the infection risk and produce model-based smooth predictive risk maps of the risk over a surface of interest [9–12]. Moreover, it allows a deeper understanding of the epidemiology and ecology of the disease [13–17]. Malaria prevalence data are likely to be spatially correlated and Bayesian geostatistical models can capture this correlation by accounting for an unobserved underlying spatial structure [18, 19]. These models are highly parameterized, and hence, model parameter estimations rely on complex algorithms such as Markov chain Monte Carlo (MCMC) sampling methods. Bayesian geostatistical methodology has been widely used for malaria risk mapping at local [20, 21], national [22], regional [23–26] and global scales [27, 28].

In Côte d’Ivoire, a country highly endemic for malaria, the national malaria prevention and control policy is based fundamentally on the use of long lasting insecticidal nets (LLINs), intermittent preventive treatment with sulfadoxine-pyrimethamine (IPT-SP) and environmental sanitation. The control strategy also includes prompt diagnosis and treatment with artemisinin-based combination therapy (ACT) [29]. No specific interventions targeting school-aged children are currently being promoted, but a recent national school-based survey in Côte d’Ivoire on parasitic diseases revealed an overall *Plasmodium falciparum* prevalence in excess of 60 % [30, 31].

The purpose of this study was to spatially analyse *P. falciparum* prevalence data obtained from the first national school-based survey on parasitic infections in Côte d’Ivoire [30–32]. A Bayesian geostatistical approach was employed and environmental and sociodemographic factors, disease prevention indicators and distance from school to the nearest health facility were considered to assess their potential effects on *P. falciparum* infection risk. Environmental factors that govern the spatial distribution of *P. falciparum* were used to produce a model-based high spatial resolution *P. falciparum* risk map for Côte d’Ivoire.

## Methods

### Study area

Côte d’Ivoire has a warm and humid climate. The average temperature ranges between 25 °C and 32 °C. There are three main seasons; warm and dry from November to March, hot and dry from March to May and hot and wet from June to October. A dense tropical moist forest covers the south-western part of the country; in the middle part of Côte d’Ivoire, a Guinean forest-savannah mosaic belt extends from east to west and a Sudanian savannah covers the northern part.

### Study design

A country-wide cross-sectional survey using the lattice plus close pairs sampling approach was designed, as described elsewhere [30–33]. In short, a grid indicating latitude and longitude at a unit of 0.5° was overlaid on a map of Côte d’Ivoire. A total of 94 schools were selected among all primary schools that comprised at least 60 children attending grades 3–5. Sixty children were sampled per school. This sample size exceeds the minimum sample size of 50 recommended by the World Health Organization (WHO) for collection of baseline information on helminth prevalence and intensity in school-aged populations within large-scale surveys [34]. The survey was carried out during the dry season from November 2011 to February 2012. When visiting the schools, geographic coordinates were recorded, using a hand-held global positioning system (GPS) receiver (Garmin Sery GPS MAP 62; Olathe, USA).

### Parasitological, demographic, prevention, treatment and socioeconomic data

To determine *P. falciparum* infection status, two drops of blood from finger prick samples were collected from enrolled children and thick and thin blood films were prepared on microscope slides. The slides were air-dried and transferred to nearby laboratories where they were stained with Giemsa and examined under a microscope by experienced laboratory technicians for *Plasmodium* species identification and parasitaemia. The number of parasitized blood cells were counted by assuming a standard white blood cell count of 8,000 per 1 μl of blood. Ten percent of the slides were randomly selected for quality control.

A pre-tested questionnaire was administered to all children participating in the survey. The questionnaire included information on household asset ownership (e.g. bicycle, fridge, radio, etc.), clinical symptoms (e.g. abdominal pain, headache, vomiting, etc.) and recent history of diseases (e.g. malaria, skin disease, schistosomiasis, etc.) [31]. Children were also asked whether they had a bed net at home, whether they slept under a bed net, whether they used other preventive measures against malaria, such as fumigating coils, insecticide spray and burning leaves and whether they took malaria treatment during the two weeks preceding the survey.

Data were double-entered and cross-checked in EpiInfo version 6 (Centers for Disease Control and Prevention; Atlanta, USA).

### Environmental data

Environmental data were obtained from satellite imagery for the year 2011. The sources and the properties of these data are summarised in Table 1. Yearly average was used for night and day land surface temperature (LST), normalized difference vegetation index (NDVI) and rainfall. The rainfall coefficient of variation was calculated by dividing the standard deviation (SD) by the mean. Land cover was grouped into three categories: (i) urban; (ii) forest/savannah; and (iii) croplands. Altitude was obtained at 1 km spatial resolution and distance to freshwater bodies was extracted from digitized maps (Health Mapper database; Geneva, Switzerland).

**Table 1:** Data sources and properties of the environmental variables used to estimate *Plasmodium falciparum* risk among school-aged children in Côte d’Ivoire

		Temporal	Temporal	Spatial
Data type	Source	resolution	coverage	resolution
Normalized difference vegetation index	MODIS/Terra^a^	16 days	2011	1 km
Day land surface temperature	MODIS/Terra^a^	8 days	2011	1 km
Night land surface temperature	MODIS/Terra^a^	8 days	2011	1 km
Land cover	MODIS/Terra^a^	Year	2011	1 km
Rainfall	ADDS^b^	10 days	2011	8 km
Rainfall coefficient of variation	Derived from rainfall	10 days	2011	1 km
Altitude	DEM^c^	–	–	1 km
Freshwater bodies	HealthMapper^d^	–	–	–

^a^Moderate Resolution Imaging Spectroradiometer (MODIS). Available at: https://lpdaac.usgs.gov/ (accessed: 1 October 2012)

^b^Africa Data Dissemination Service (ADDS). Available at: http://earlywarning.usgs.gov/fews/datadownloads/Continental%20Africa/Dekadal%20RFE (accessed: 1 October 2012)

^c^Digital elevation model (DEM). Available at: http://eros.usgs.gov/ (accessed: 1 October 2012)

^d^HealthMapper database. Available at: http://health-mapper-release-5.software.informer.com/ (accessed: 1 October 2012)

### Statistical analysis

Children were grouped into two age categories: (i) 5–10 years and (ii) 11–16 years. Distance from school to the nearest health facility was obtained from the “Programme National de Santé Scolaire et Universitaire” (PNSSU) and was summarised into three categories, i.e. (i) < 1 km, (ii) 1–5 km, and (iii) > 5 km. The first category included schools located in villages or towns with a health facility. For assessment of socioeconomic status, an asset-based approach was used to stratify children into five socioeconomic groups [31].

Bayesian geostatistical stochastic search variable selection (SSVS) was performed to explore all possible models within a geostatistical framework and select the most important predictors for *P. falciparum* infection [35, 36]. In addition, a parameter expanded normal mixture of inverse gamma (peNMIG) prior parameterization was used to address the oversampling of the categorical variables around zero that can arise with more traditional parameterizations [37]). Details related to the variable selection procedure are provided in Additional file 1.

A first variable selection that included demographic (age and sex) and environmental variables was performed to build a predictive model of the *P. falciparum* infection risk across Côte d’Ivoire (variable selection 1). In a second step, variables at individual level about prevention, treatment, distance from school to the nearest health facility and socioeconomic status, which were not available at 1×1 km spatial resolution, were also explored for selection in order to assess their effect on *P. falciparum* infection risk (variable selection 2). The possible nonlinear relationships of variables were taken into account by considering the inclusion of categorical predictors in the model. We built both selection procedures to enable the model to choose the best functional form of each predictor, i.e. categorical or linear. With the exception of age, and distance from school to the health facilities that were categorised, predictors were introduced in both functional forms in the model for the variables, which could be expressed either as categorical or linear. The variables and their functional form included in the final models were the ones with posterior probability of inclusion over 50 % (median probability model).

Geostatistical logistic regression models within a Bayesian framework of inference were performed to analyse the risk of infection with *P. falciparum* via MCMC simulation algorithms for estimation of model parameters. Let *Y*_*ij*_ be the *P. falciparum* infection status for child *j* (*j* = 1, …, *J*) in school *i* (*i* = 1, …, *I*). We assumed that *Y*_*ij*_ arises from a Bernoulli distribution with probability *p*_*ij*_ such as, *Y*_*ij*_ ~ Be (*p*_*ij*_). We modelled covariates *X*_*ij*_ and school-specific spatial random effects *φ*_*i*_ on the logit scale, i.e. log*it* (*p*_*ij*_) = *X*_*ij*_*β* + *φ*_*i*_, where *β* represents the vector of regression coefficients, including a constant. Spatial random effects were assumed to follow a multivariate normal prior distribution, *φ* ~ MVN (0, Σ). The variance-covariance matrix Σ introduced spatial dependency through an isotropic exponential correlation function of distance between locations as follows: Σ_kl_ = σ^2^ exp (−*ρd*_*kl*_), where *d*_*kl*_ is the Euclidian distance between a pair of schools *k* and *l*, the variance σ^2^ measures the spatial geographic variability and *ρ* is a parameter that controls the rate of correlation decay. The range, defined as the minimum distance at which spatial correlation between locations is below 5 %, is calculated as 3/ρ. To complete model specification, prior distributions were assigned to model parameters. For the regression coefficients, non-informative normal prior distributions were chosen such as β ~ N (0, 0.01) where β = (*β*_*1*_,… *β*_*K*_)^T^. For the variance and the correlation decay, inverse gamma and gamma distributions were respectively assumed, i.e. σ^2^ ~ IG (2.01, 1.01) and ρ ~ G (0.01, 0.01). Model prediction was done using Bayesian kriging [38]. We assessed model convergence by visual examination of history and density plots. A sample of the last 500 iterations were stored for prediction on a grid of 352,911 pixels with a spatial resolution of 1 km.

To validate our model, a geostatistical logistic regression model was fitted on a sub-dataset of 73 randomly selected schools (around 80 % of the number of schools in the original dataset). We then predicted the risk at the remaining schools and compared our predictions with the observed prevalence data. Model predictive ability was assessed by calculating the mean absolute error (MAE), which is the mean of the absolute differences between the median of the predicted *P. falciparum* infection risk and the observed prevalence. Model uncertainty was assessed by the sum of the standard deviations (SDs) of the predictive distributions.

### Implementation details

Geostatistical variable selection and model fit were implemented in OpenBUGS v.3.2.2 (University of Helsinki; Helsinki, Finland). Fortran 95 (Compaq Visual Fortran Professional version 6.6.0, Compaq Computer Corporation; Houston, USA) was used for prediction. STATA version 10 (Stata Corporation; College Station, USA) was used for data management and preliminary analysis. ArcGIS version 10.0 (Environmental Systems Research Institute Inc; Redlands, USA) was used to display study results on maps.

## Results

**Fig. 1:**
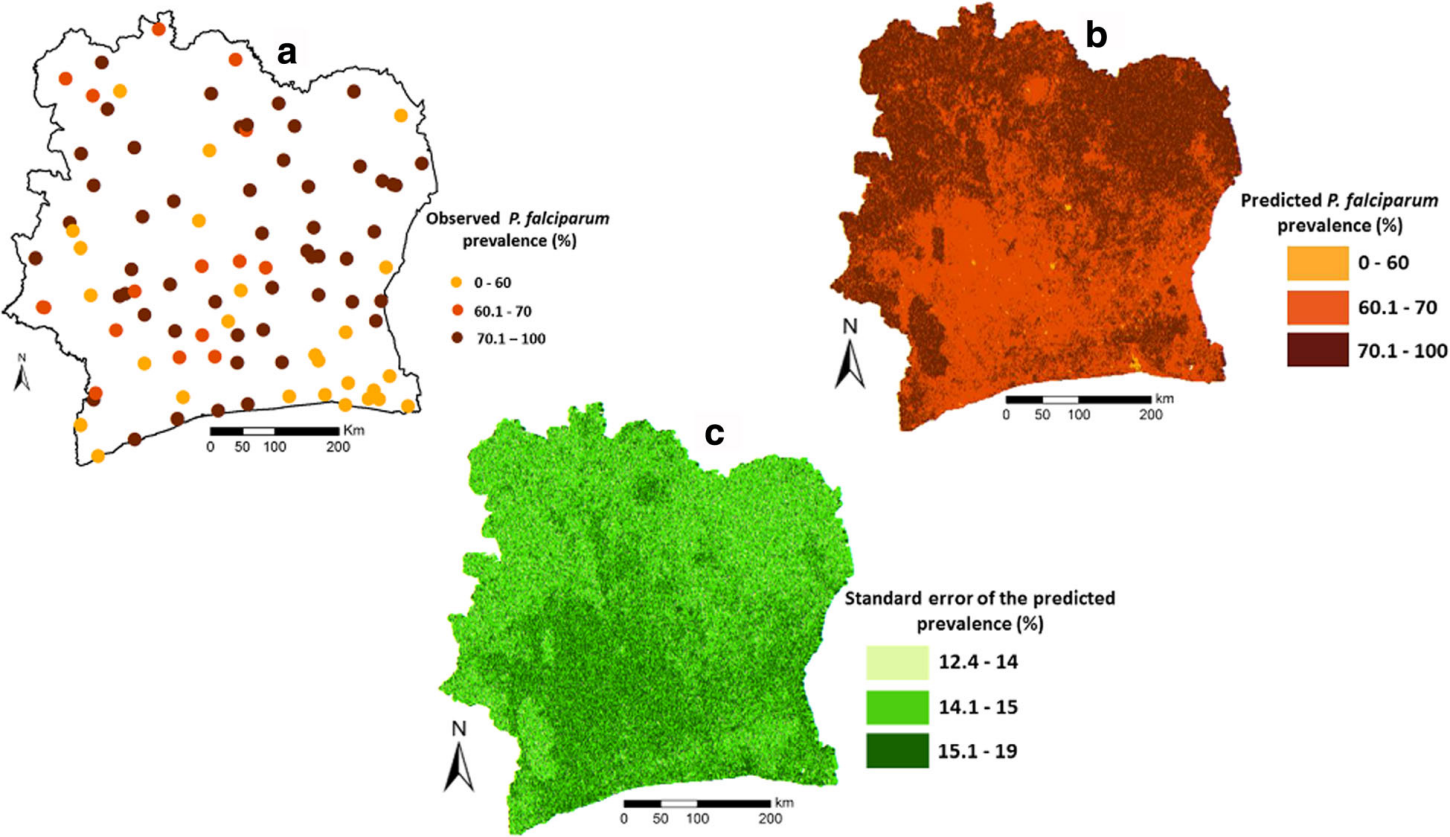
Observed (**a**), predicted (**b**) and standard error of the predicted (**c**) *Plasmodium falciparum* infection prevalence among school-aged children in Côte d’Ivoire. Data used for prediction were obtained from a national cross-sectional survey carried out in 93 schools in the dry season between November 2011 and February 2012. Model-based predictions, including standard errors of predictions, were done within a Bayesian geostatistical framework

Complete data records were available from 5,322 children aged 5–16 years in 93 schools. Of note, one of the sampled schools refused to participate. The prevalences of *P. malariae* and *P. ovale* were very low; 3.7 % and 0.3 %, respectively. *P. falciparum* was the predominant species with an overall observed prevalence of 69.2 %. All subsequent analyses focussed on *P. falciparum* only. The spatial distribution of the observed *P. falciparum* infection prevalence is shown on map A in Fig. 1.

We assessed potential correlation between predictors in a preliminary analysis, but none were considered as highly correlated, since the absolute value of the Pearson’s correlation coefficient never exceeded 0.9 (*r* < 0.9). Hence, all of them were considered for selection as potential predictors of malaria risk. Median probability models with their posterior probability, as well as posterior inclusion probability of each predictor for both variable selection procedures are shown in Table 2. For variable selection 1, where only demographic and environmental predictors were explored, a posterior inclusion probability superior to 50 % was obtained for land cover. However, when we additionally included prevention, treatment and socioeconomic data and distance from school to the nearest health facility in the variable selection procedure (variable selection 2), only socioeconomic status was retained as an important predictor.

**Table 2:** Geostatistical variables selection results

	Variable selection 1		Variable selection 2	
Predictors	Median probability model	Predictor posterior inclusion probability	Median probability model	Predictor posterior inclusion probability
Age	0	2.4 %	0	1.6 %
Gender	0	27.6 %	0	24 %
Altitude				
Linear	0	1.6 %	0	1.5 %
Categorical	0	4.2 %	0	2.4 %
Distance to freshwater bodies				
Linear	0	0.9 %	0	0.6 %
Categorical	0	5.9 %	0	2.4 %
Rainfall				
Linear	0	2.2 %	0	2.3 %
Categorical	0	25.0 %	0	14.8 %
Rainfall coefficient of variation				
Linear	0	1.9 %	0	2.1 %
Categorical	0	9.8 %	0	20.6 %
Normalized difference vegetation index				
Linear	0	1.2 %	0	0.5 %
Categorical	0	4.1 %	0	4.0 %
Night land surface temperature				
Linear	0	2.8 %	0	1.0 %
Categorical	0	9.0 %	0	1.5 %
Day land surface temperature				
Linear	0	1.3 %	0	0.9 %
Categorical	0	1.7 %	0	3.0 %
Land cover	×	51.1 %	0	19.1 %
Bed net ownership			0	2.1 %
Fumigating coil			0	13.4 %
Insecticide spray			0	15.5 %
Smoke by burning leaves			0	3.3 %
Children sleeping under a net			0	1.1 %
Children slept under a net last night			0	1.8 %
Malaria treatment			0	3.5 %
Distance to nearest health facility			0	3.3 %
Socioeconomic status			×	100 %

Variable selection 1 included only demographic and environmental variables. Variable selection 2 additionally included prevention and socioeconomic variables. × (selected), 0 (not selected); Median probability model is presented together with posterior inclusions probability of the predictors

Estimates of model parameters and model validation results for the Bayesian geostatistical logistic regression model with land cover as predictor are summarised in Table 3. Croplands and forest/savannah were positively associated to *P. falciparum* infection compared to urban setting (croplands odds ratio (OR): 1.95, 95 % Bayesian credible interval (BCI): 1.23–3.03); forest/savannah OR: 2.30, 95 % BCI: 1.43–3.81). The spatial range was 285 km (95 % BCI: 139–477 km), indicating important residual spatial correlation. Model validation showed that the model predicts a random sample of 20 % of the data with a MAE of 0.11 and a sum of SD of the posterior predictive distribution of 1.81.

**Table 3:** Parameter estimates of two Bayesian geostatistical models with environmental and socioeconomic predictors

Parameter	Model 1		Model 2	
	OR	95 % BCI	OR	95 % BCI
Land cover				
Urban	1.00			
Croplands	1.95	(1.23; 3.03)^a^		
Forest/savannah	2.30	(1.43; 3.81)^a^		
Socioeconomic status				
Poorest			1.00	
Poor			0.77	(0.61; 0.96)^a^
Middle			0.66	(0.53; 0.84)^a^
Wealthier			0.48	(0.39; 0.59)^a^
Wealthiest			0.44	(0.35; 0.56)^a^
	Median	95 % BCI	Median	95 % BCI
Spatial variance σ^2^	0.46	(0.29; 0,80)^a^	0.41	(0.24; 0,75)^a^
Range (km)	285	(139; 471)	259	(123; 457)
	Predictive ability			
MAE	0.11			
Sum of SD	1.81			

^a^Important covariate based on 95 % BCI

Model 1: Bayesian geostatistical model with environmental predictor

Model 2: Bayesian geostatistical model with socioeconomic predictor

*Abbreviations: OR* odds ratio, *95 % BCI* lower and upper bound of a 95 % Bayesian credible interval, *MAE* mean absolute error, *Sum of SD* sum of standard deviation

Estimates of model parameters for the Bayesian geostatistical logistic regression with socioeconomic status are also presented in Table 3. Socioeconomic status showed a significant association with the risk of *P. falciparum* infection; the wealthier the household, the lower the risk for *P. falciparum* infection. A substantial residual spatial correlation of 259 km (95 % BCI: 123–457 km) was estimated.

Map B in Fig. 1 illustrates the smooth map of the estimated *P. falciparum* infection prevalence among school-aged children in Côte d’Ivoire. Lowest prevalences (around 45 %) were estimated for small urban aggregated areas in the south-east of the country, close to Abidjan, central-southern and central-western parts. Prevalences above 70 % were found in the north, central-east, south-east, west and south-west of Côte d’Ivoire. Map C in Fig. 1 shows the standard error of the predicted *P. falciparum* infection prevalence. High prediction errors were mostly found from central-west to central-east of the country.

## Discussion

The purpose of this study was to (i) identify sociodemographic, environmental and disease prevention indicators associated with *P. falciparum* infection prevalence and (ii) produce a smooth risk map of *P. falciparum* infection among school-aged children for Côte d’Ivoire. To our knowledge, this spatial analysis is the first at the national level based on *P. falciparum* data collected within a few weeks during the dry season in late 2011/early 2012. The results obtained from this spatially explicit analysis are useful for current and future malaria control efforts in Côte d’Ivoire.

Among the environmental covariates, only land cover was selected by the geostatistical variable selection procedure. Precipitation, temperature and distance to freshwater bodies - factors that have previously been associated to malaria risk in Côte d’Ivoire [21, 22] - were not identified as important predictors in the current investigation. Possible explanations arise from the use of different data sources; while the national survey reported here was conducted in the dry season, the aforementioned study pursued at country level [22] used historical data obtained over a period of 20 years and during different periods of the year. In addition, particular geographic patterns such as for the mountainous area in western Côte d’Ivoire [21], where particular climatic and environmental conditions prevail and scale differences across studies may further explain contrasting results. Regarding the distance to water body, this variable had not been selected by our modelling framework as a potential risk factor for malaria. This observation might be explained by the fact that the distance from school to water body is not precise enough to capture this effect or that the source of water body is not sufficiently detailed. Unfortunately, we could not afford to collect information on the distance from each participant’s home to the nearest water body. Further effort is needed to identify additional water body information in Africa.

The spatial model with land cover as covariate indicated that school-aged children living in areas with forest/savannah and croplands are at higher risk of *P. falciparum* compared to those from urban areas. On one hand, this result suggests that the endemicity of malaria in Côte d’Ivoire is linked to two main vegetation types, namely the forest in the south and the savannah in the north, which basically do characterise the two main ecozones of the country. In contrast, other studies using land cover showed that the forest area was associated with a decrease of malaria incidence [39] or risk [40]. It is important to underline that the south of Côte d’Ivoire is mainly characterised by tropical rainforest in which people are living and this pattern might differ in other studies. On the other hand, people living in urban areas generally have better access to treatment and prevention measures, compared to rural areas, which is reflected by our results.

Interestingly, the second geostatistical variable selection that included environmental variables, prevention and distance from school to the nearest health facility suggested that only socioeconomic status, as assessed by the wealth index, explained *P. falciparum* infection risk. Wealthier households were associated with a low risk of *P. falciparum* infection and is line with results from other studies [3, 41, 42]. Basically, this result is consistent with the first variable selection procedure, where only environmental factors were considered. Indeed, the urban area is characterised by overall higher socioeconomic status compared to the forest/savannah and cropland areas. Of note, Côte d’Ivoire is still mainly rural, although urbanisation progresses rapidly [20, 21, 31, 43]. As shown elsewhere, *P. falciparum* infection and parasitaemia are positively associated with low socioeconomic status.

In the present study employing recent epidemiological data, very high prevalences of *P. falciparum* (> 70 %) were found in the entire north and the south-west (Taï forest region) of Côte d’Ivoire. This is in contrast with lower prevalences obtained from a previous spatial analysis using historical data focussing on children aged < 16 years [22]. Recent environmental transformations, such as rice farming in the north [44] and progression of deforestation in the Taï forest in the south-west, led to increased population densities [45, 46], which might explain the change in *P. falciparum* prevalence rates in these areas. However, differences in survey designs and large heterogeneities of historical data must be considered to deepen the understanding of potential changes in *P. falciparum* prevalence and parasitaemia in space and time [47]. The design of the present study allowed us to have more uniformly distributed data across the country than before, and hence, prediction uncertainty was minimized.

## Conclusion

This study provided a comprehensive analysis on the spatial distribution of *P. falciparum* infection among school-aged children across Côte d’Ivoire. Given the high burden of *P. falciparum* infections in the school-aged population, there is a need for intervention strategies that also target this age group. Notwithstanding, since the end of 2014, the national malaria control programme in Côte d’Ivoire is making LLINs available to the entire population, including school-aged children, thus an important step is taken to tackle the malaria burden in this specific age group. The produced smooth *P. falciparum* prediction map, in conjunction with uncertainty estimates, represent useful tools for scaling up current and future malaria control interventions. Future predictive risk profiling should include other factors such as population density and more detailed information on intervention coverage in order to better understand the impact of ongoing malaria interventions.

## Additional file

Additional file 1: Bayesian geostatistical stochastic search variable selection. (DOCX 22 kb)

## Abbreviations

ACT: Artemisinin-based combination therapy
BCI: Bayesian credible interval
GIS: Geographic information system
GPS: Global positioning system
IG: Inverse-gamma
IPT-SP: Intermittent preventive treatment with sulfadoxine-pyrimethamine
LLIN: Long lasting insecticidal net
LST: Land surface temperature
MAE: Mean absolute error
MCMC: Markov chain Monte Carlo
NDVI: Normalized difference vegetation index
OR: Odds ratio
peNMIG: parameter expanded normal mixture of inverse gamma
PNSSU: “Programme National de Santé Scolaire et Universitaire”
SD: Standard deviation
SSVS: stochastic search variable selection
WHO: World Health Organization
YLDs: Years lived with disability
