# Supplementary material for: Spatial mapping and prediction of Plasmodium falciparum infection risk among school-aged children in Côte d’Ivoire
Source: Parasit Vectors. 2016 Sep 7;9(1):494. doi: 10.1186/s13071-016-1775-z (PMC5015250; doi:10.1186/s13071-016-1775-z)
Supplement: Additional file 1: — Bayesian geostatistical stochastic search variable selection. (DOCX 22 kb) [file 13071_2016_1775_MOESM1_ESM.docx]

**Additional file 1: Bayesian geostatistical stochastic search variable selection**

We used a stochastic search variable selection [1] within a geostatistical framework for selection of predictors to include in our model, while taking into account the spatial correlation in the data. In addition, we used a parameter expanded normal mixture of inverse gamma (peNMIG) distribution [2, 3] for the regression coefficients to allow selection between categorical covariates in blocks and linear covariates. Thus, for each category *l* (*l* = 1, …, *L*; excluding baseline) of categorized covariates $k_{1}$ ($k_{1}=1, \ldots, K_{1}$), we defined regression coefficients$\beta_{k_{1}l}$ as the product of an overall contribution ($o_{k_{1}}$) and a category-specific effect ($s_{k_{1}l}$), such as $\beta_{k_{1}l}=o_{k_{1}}s_{k_{1}l}$. For linear covariates $k_{2}$ ($k_{2}=1, \ldots, K_{2}$), the specific effect *s* was set to 1 and$\beta_{k_{2}}=o_{k_{2}} .$ Indicators $I_{k_{1}}$ and $I_{k_{2}}$indicate the presence or absence of the corresponding variable in the model at the level of the variance of the overall contribution. In particular, the variance of the overall contribution was shrunk to 0 when the variable was excluded from the model or stayed non-informative when the variable was included in the model. In addition, a binary indicator *Ind_k_* that multiplies the regression coefficient of variable $k$ (k$=1, \ldots, K$), irrespective of its functional form, was introduced to determine whether predictor $k$is selected under its linear form (*Ind_k_*=1) or under its categorical form (*Ind_k_*=0).

We considered the following prior distributions for model parameters: 1) a Bernoulli distribution for the indicator; $I_{k_{1}}\sim Be\left( w_{k_{1}} \right)$, $w_{k_{1}}\sim Beta\left( 4, 2 \right),$ $I_{k_{2}}\sim Be\left( w_{k_{2}} \right)$, $w_{k_{2}}\sim Beta\left( 4, 2 \right)$, ${Ind}_{k}\sim Be\left( w_{k} \right)$, $w_{k}\sim Beta\left( 4, 2 \right)$, 2) a mixture of two Gaussian distribution for the category-specific effect that shrunk the effect towards $\left| 1 \right|$ (multiplicative identity); $s_{k_{1}l}\sim N\left( m_{k_{1}l},1 \right)$, $m_{k_{1}l}\sim\frac{1}{2}\delta_{1}\left( m_{k_{1}l} \right)+\frac{1}{2}\delta_{-1}\left( m_{k_{1}l} \right),$ and 3) a normal prior distribution for the overall contribution that shrunk it to 0 when the variable $k_{1}$ was excluded from the model ($I_{k_{1}}=0$) and staid vague when the variable $k_{1}$ was included in the model ($I_{k_{1}}=1$);,$o_{k_{1}}\sim N(0,\tau^{2})$, $o_{k_{2}}\sim N(0,\tau^{2})$, $\tau^{2}\sim I_{k_{1}}IG\left( 5, 25 \right)+\left( 1-I_{k_{1}} \right) 0.00025*IG(5, 25)$.

A Gibbs sampler chain for 150,000 iterations was used to run both geostatistical variable selections and the last 5,000 estimates of each indicator ${Ind}_{k,}I_{k_{1}}$ and $I_{k_{2}}$were used to calculate the posterior inclusion probabilities. Linear predictors were included if ${Ind}_{k}I_{k_{1}}=1$, while categorized predictors were included if $\left( 1-{Ind}_{k} \right)I_{k_{2}}=1$. Such variable selection explores all possible models and the median probability model defined the final model and included all predictors with a posterior inclusion probability superior to 50%.

**References**

1. George EI, McCulloch RE: **Variable selection via Gibbs sampling**. *J Am Stat Assoc* 1993., **88**:881–889.

2. Scheipl F, Fahrmeir L, Kneib T: **Spike-and-slab priors for function selection in structured additive regression models**. *J Am Stat Assoc* 2012, **107**:1518–1532.

3. Chammartin F, Hürlimann E, Raso G, N’Goran EK, Utzinger J, Vounatsou P: **Statistical methodological issues in mapping historical schistosomiasis survey data**. *Acta Trop* 2013, **128**:345-352.
